# Spontaneous hepatic rupture during late pregnancy in a patient with solitary necrotic nodule of the liver: A case report

**DOI:** 10.3389/fmed.2022.936006

**Published:** 2022-10-20

**Authors:** Jilong Wang, Qilin Yi, Ya Guo, Tao Peng

**Affiliations:** Department of Hepatobiliary Surgery, The First Affiliated Hospital of Guangxi Medical University, Nanning, Guangxi, China

**Keywords:** spontaneous hepatic rupture, solitary necrotic nodule of the liver, pregnancy, case report, multidisciplinary collaboration

## Abstract

**Background:**

Spontaneous hepatic rupture (SHR) during pregnancy is a rare and life-threatening event, which usually occurs together with preeclampsia, eclampsia, HELLP syndrome, or liver tumors. However, SHR resulting from solitary necrotic nodule of the liver (SNNL) is extremely rare.

**Case presentation:**

We report the case of a 32-year-old pregnant woman who presented at 33 weeks of gestation with abdominal pain and emesis. Transabdominal ultrasound and magnetic resonance imaging revealed massive hemoperitoneum and lesions in the left lobe of the liver. An emergency cesarean section was performed and the hepatic rupture was managed surgically *via* left lateral lobectomy. The postprocedural course was uneventful. The premature baby successfully survived, and the patient was discharged 8 days after admission without complications. Histological examination revealed a diagnosis of SNNL, which resulted in the hepatic hematoma and SHR.

**Conclusion:**

To our knowledge, this is the first case of SHR resulting from SNNL during late pregnancy. Multidisciplinary collaboration and surgical management are important cornerstones for improving the perinatal outcomes when SHR is suspected in a pregnant patient.

## Introduction

Spontaneous hepatic rupture (SHR) in pregnancy is a rare and life-threatening complication, which carries a high maternal and perinatal mortality (1). It is usually associated with preeclampsia, eclampsia, HELLP syndrome or liver tumors (2). However, SHR resulting from solitary necrotic nodule of the liver (SNNL) is extremely rare. SNNL is a rare benign non-neoplastic lesion of the liver, first reported by Shepard and Lee in 1983 (3). It has no definite clinical manifestations, and is easily misdiagnosed as liver primary or metastatic tumor. Most patients are accidentally found during physical examination and seek further treatment. The etiology of SNNL remains uncertain, and surgical resection is required for pathological examination to confirm the diagnosis. Pathologic features of SNNL are a central necrotic core surrounded by a fibrotic membrane infiltrated by inflammatory cells, including histocyte, lymphocytes, plasma cells, eosinophils, and so forth (4).

Herein, we report a case with a rare occurrence of SHR from SNNL at late pregnancy, and both the pregnant woman and her newborn survived after surgery.

## Case presentation

A 32-year-old primigravida at 33 weeks gestation was referred to our Emergency Department from a county-level hospital with a three-day history of abdominal pain and emesis. The patient was complaining of a continuous and worsening epigastric pain, with intermittent vomiting. She was evaluated in her local hospital: the vital parameters were all normal, but blood tests evidenced hemoglobin (Hb) 75.0g/dL. Ultrasound (US) showed hepatic hematoma in the left lobe and free abdominal fluid. The fetal heart sound was normal and there were no images suggesting placental abruption. She was transfused with 2 units of red blood cells (RBC) and then transferred to our hospital.

The patient had a smooth antenatal course and no history of trauma or intercourse prior to hospital admission. Two years ago, she was diagnosed with a left lateral hepatic hemangioma, approximately 1.5 cm in diameter, but didn’t receive systematic treatment. She had no antecedent of oral contraceptives use, and denied alcohol or drug abuse, too. Physical examination revealed that the pregnant woman was pale but had stable vital signs: blood pressure of 115/69 mmHg, heart rate of 86 beats/min, and body temperature of 37.0°C. The uterine fundus was three fingers above the umbilicus without contractions, and the fetal heart rate was 146 beats/min. Laboratory tests showed Hb at 83.7 g/L, hematocrit at 25.1%. Serological tests for liver enzymes, hepatitis B and C, and tumor markers were normal, except for alpha-fetoprotein (AFP) of 206.29 ng/mL, carbohydrate antigen of 125 (CA125) 110.4 U/mL and PIVKA-II of 52.31 mAU/ml.

**FIGURE 1:**
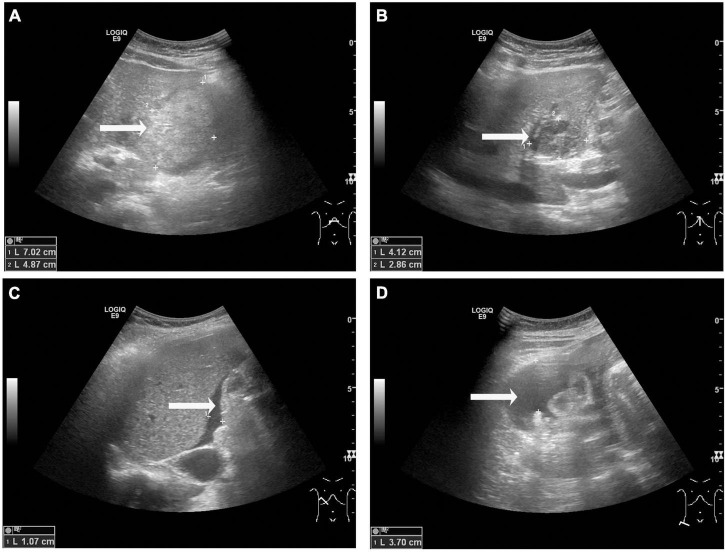
Transabdominal ultrasound observations at admission. **(A)** A heterogenic area was observed on the visceral surface of the left hepatic lobe, measuring 7.0 cm × 3.8 cm × 4.9 cm (arrow). **(B)** A hypoechoic area was observed in segment III of the liver, measuring 4.1 cm × 3.1 cm × 2.9 cm **(B,** arrow). **(C,D)** Fluid dark areas were detected in perihepatic **(C,** arrow) and right lower abdominal cavity **(D,** arrow).

The US performed by the gynecologist evidenced that the placenta and fetal heart sound were normal. Transabdominal US showed a 7.0 cm × 3.8 cm × 4.9 cm heterogenic lesion with well-defined margins on the visceral surface of the left hepatic lobe (Figure 1A), which was considered as hematoma. There was a hypoechoic mass in segment III measuring 4.1 cm × 3.1 cm × 2.9 cm (Figure 1B), considering the possibility of hepatic hemangioma. Meanwhile, fluid dark areas were detected in perihepatic and right lower abdominal cavity (Figures 1C,D), which was confirmed by ultrasound-guided abdominal paracentesis as old non-coagulant blood.

**FIGURE 2:**
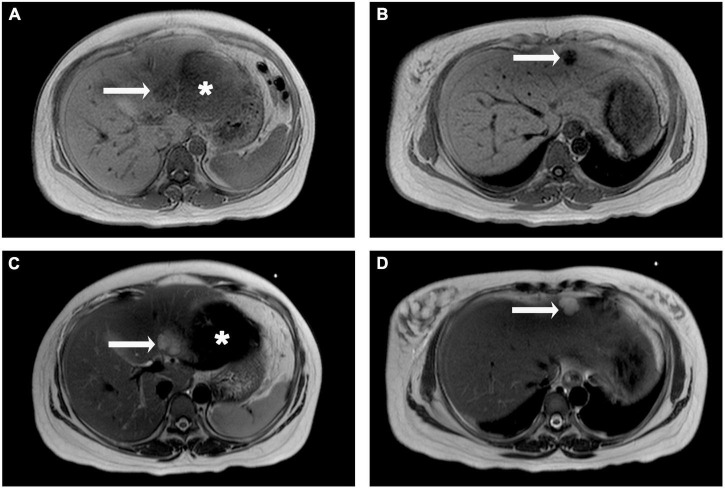
Liver magnetic resonance imaging (MRI) plain scan observations. The lesions in segment III and II of the liver were hypointense on T1-weighted images **(A,B,** arrow), but hyperintense on T2-weighted image **(C,D,** arrow). A large area of slightly longer T1 and shorter T2 signals is seen below the left hepatic lobe **(A,C,** asterisk).

The patient and her family refused to performed computed tomography (CT) or enhanced magnetic resonance imaging (MRI) examination for fear of adverse effects on the fetus, and finally had to undergo the emergency abdomen MRI plain scan. Two lesions were found in the left lateral lobe of liver, which were hypointense in T1-weighted images (Figures 2A,B) and subtle hyperintensity in T2-weighted images (Figures 2C,D), indicating a high possibility of hemangioma. Moreover, there was a large hematoma on the visceral surface of the left hepatic lobe with longer T1 and shorter T2 signals (Figures 2A,C).

**FIGURE 3:**
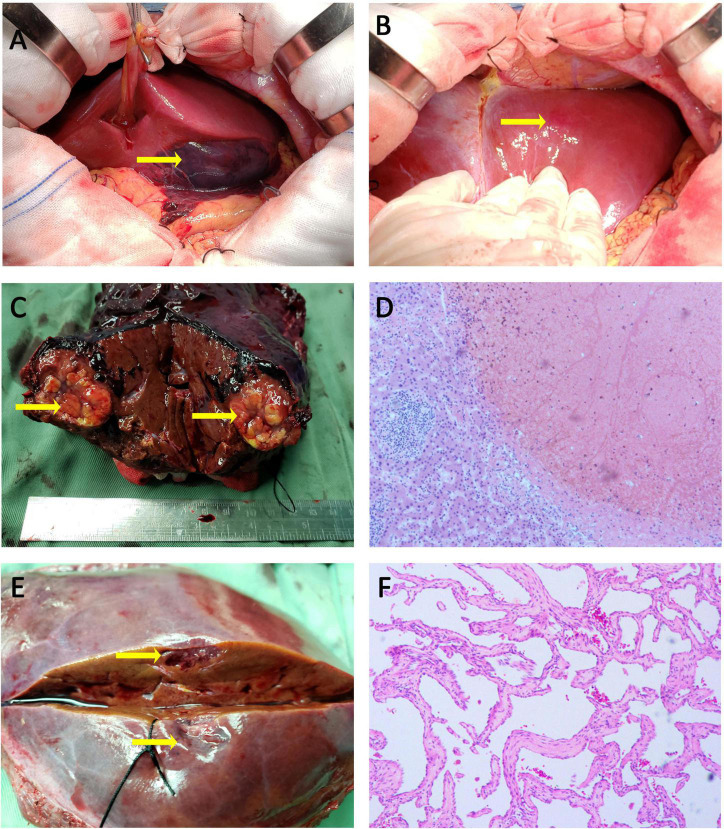
Intraoperative photographs, specimen photographs and Pathological examination. **(A)** A subcapsular hemotoma with a marked laceration was found on the visceral surface of the left hepatic lobe. **(B)** A hepatic hemangioma was observed on the surface of segment II. **(C)** Surgical specimen demonstrated a rounded, grayish-yellow, and non-encapsulated nodule with well-defined margins under hemotoma. **(D)** The lesion under hepatic hemotoma was SNNL, characterized as the central necrotic tissue surrounded by a fibrous capsule containing inflammatory cells (H&E, × 100). **(E)** The appearance of cross-sectional surface for lesion in segment II was grayish-brown. **(F)** The lesion in segment II was demonstrated to be hepatic hemangioma (H&E, × 100).

Considering the fetus’ safety and the risk of continued bleeding in the patient, emergency surgical treatment was decided after a multidisciplinary discussion with colleagues in obstetrics, anesthesiology and neonatology. First, cesarean section was performed by obstetricians, and a live female infant weighing 2750 g was delivered. The Apgar score was 7 points at 1 min and 10 points at 5 min. Then the premature baby was sent to the pediatric intensive care unit for further treatment. Since no evidence of placental abruption or active intrauterine bleeding was detected, an exploratory laparotomy was performed with a reverse L incision after the uterus was sutured. A large ruptured hematoma on the visceral surface of the left lateral lobe of liver was evident with small amount of active bleeding (Figure 3A). Meanwhile, a hemangioma on the diaphragmatic surface of the hepatic segment II was also found (Figure 3B). About 1000 ml of blood clot was removed from the abdominal cavity. Then the left lateral lobectomy was performed and 4 units of RBC and 600 mL plasma were transfused during operation. The abdomen was then closed. When the specimen was dissected, a rounded, 4 cm in diameter, grayish-yellow and non-encapsulated nodule with well-defined margins was observed under the hematoma (Figure 3C). This nodule was demonstrated to be SNNL by histological examination, characterized as the central necrotic tissue surrounded by a fibrous capsule containing inflammatory cells (Figure 3D).

Meanwhile, a 1.5 cm grayish-brown lesion was found on the surface of segment II (Figure 3E), which was pathologically confirmed as a hepatic hemangioma (Figure 3F).

The patient had an uneventful postoperative clinical course while hospitalized, and was discharged after 8 days from admission in good physical condition without post-operative complications.

## Discussion

Since first described by Abercrombie in 1844, more than 200 cases of SHR during pregnancy has been reported in the world literature (1, 5). SHR represents mainly a postpartum complication, its incidence ranged from 1 in 45,000 to 1 in 225,000 deliveries (1). A recent literature review of SHR revealed a maternal mortality rate of 39% (6), and a perinatal mortality rate of 44% (7). Because the signs and symptoms of hepatic bleeding are non-specific, physicians should be alert to the possibility of SHR when pregnant women suddenly developed unexplained, severe abdominal pain, shoulder pain and vomiting. Emergency US, CT or MRI should be performed to confirm the diagnosis in a timely manner (6, 8).

**FIGURE 4:**
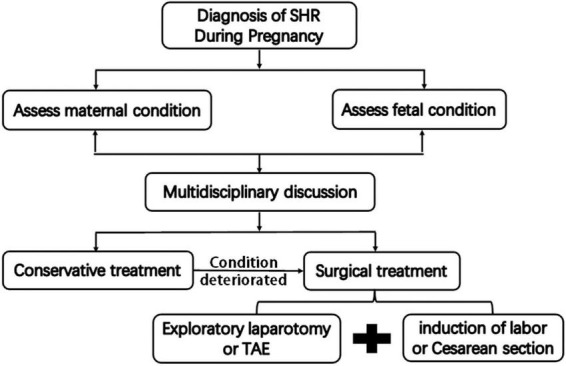
Flow diagram for management of spontaneous hepatic rupture during pregnancy.

Once a definite diagnosis of SHR is made in pregnancy, immediate action should be taken (Figure 4). The standard management of patients with SHR in pregnancy includes emergency the induction of labor or cesarean section followed by immediate exploratory laparotomy. At this point, different procedures can be performed depending on intraoperative conditions, including hepatorrhaphy with a dedicated hemostatic device, perihepatic packing, and hepatectomy. If the condition permits, hepatic artery embolization may even be attempted to stop the bleeding. Sometimes, conservative non-operative management could be attempted for highly selected patients. However, we believe that conservative treatment is inapplicable in this case, because the patient had a large amount of blood in the abdominal cavity and the potential for further bleeding, which threatened the life of the fetus. Therefore, we recommended the use of aggressive intervention in the form of an emergency cesarean section with transcatheterarterial embolization (TAE) of the hepatic artery or exploratory laparotomy for the first-line treatment in this case after a multidisciplinary discussion. However, the patient and his family refused TAE since the possibility of malignant liver lesions could not be ruled out. Therefore, we performed an exploratory laparotomy after cesarean section by the obstetrician. Since both the bleeding point and the hemangioma were located in the left external lobe, we performed a left external lobectomy, which successfully controlled the bleeding and removed the lesions.

Spontaneous hepatic rupture (SHR) during pregnancy is usually associated with pregnancy-induced hypertensive disorders, such as severe preeclampsia, eclampsia, or HELLP syndrome, and sometimes primary or metastatic liver tumors (8, 9). It can also occur in otherwise healthy pregnant women without obvious cause but is extremely rare. In this study, we confirmed that the nodule under the hematoma was SNNL, which may have caused the SHR in this pregnant woman. SNNL is a well-known but rarer mostly benign lesion, about 60 cases have been reported so far, but none have been reported in pregnant women (10). Most are discovered incidentally through radiologic examination, surgery, or autopsy, which is more common in elderly men, and generally occurs in the right liver lobe. Three-quarters of the cases have a diameter of less than 20 mm (3). It is difficult to distinguishing SNNL from intrahepatic space occupying disease due to its low incidence, as well as insidious onset, lack of specific manifestations and clinical indicators (10). Most lesions have been identified pathologically after surgical resection. Preoperative imaging examination to monitor the lesion is of great importance. SNNL usually presents as heterogeneous hypoechoic nodules on ultrasonography with unclear edges (11). Enhanced MRI is helpful to distinguish SNNL from other lesions. SNNL appears as low signal in T1-weighted image, and high-intensity in T2-weighted image (12). Studies have shown that when the MRI examination time was extended to 1 h, SNNL lesions appear as moderate/significant enhancement in the periphery and low internal intensity (13). SNNL usually shows uniform density on plain CT scan, but low density with peripheral enhancement at arterial phase of enhancement. MRI seems superior to CT in the discovery of liquefaction necrosis (14). Although the ultrasound and MRI images of this patient are consistent with previous literature descriptions, these findings are not yet sufficient to confirm SNNL, and the only reliable diagnosis is histological examination of the entire lesion (15). Histopathologically, the SNNL usually shows that the central necrotic area is surrounded by a dense transparent fibrous capsule in the outer layer. The necrosis may be liquefied necrosis, coagulation necrosis or mixed necrosis (15). The pathological HE staining of this patient shows that the central necrotic tissue is surrounded by a border zone, which contains a small amount of mononuclear inflammatory cells and fibrotic changes, and the outer layer shows normal hepatocytes.

Although the etiology of SNNL is currently uncertain, it includes trauma-related diseases, underlying hemangioma or parasitic infection (10, 16). It has been hypothesized that SNNL are likely to be the presence of “vasa vasorum”, evolving from a small hemangioma (17). In this report, the patient has a history of hepatic hemangioma, and we suspect that the presence of hepatic hemangioma promoted the occurrence of SNNL, while hepatocyte necrosis and inflammation promoted the formation of subcapsular hematoma, thus leading to the occurrence of SHR (2, 18). Currently, the best treatment for SNNL is surgical resection. Fortunately, under the early imaging examination and multidisciplinary consultation, the treatment plan of simultaneous cesarean section and exploratory laparotomy were made promptly, and finally ensured the feat of both maternal and neonatal survival.

To the best of our knowledge, this is the first case of SHR resulted from SNNL during pregnancy, with a favorable outcome for both the mother and the newborn. Multidisciplinary collaboration and timely surgical management are important cornerstones for improving the perinatal outcomes when SHR is suspected in a pregnant patient.

## Data availability statement

The original contributions presented in this study are included in the article/supplementary material, further inquiries can be directed to the corresponding author.

## Ethics statement

This study was approved by the First Affiliated Hospital of Guangxi Medical University Ethical Review Committee [2021(KY-E-011)]. Written informed consent was obtained from the patient(s) for the publication of this case report.

## Author contributions

JW and TP conceived and designed the study. JW and QY collected the patient’s clinical data and drafted the manuscript. YG and TP critically commented on and revised the manuscript. All authors provided final approval of the version that was submitted.
